# Activation of STING by cGAMP Regulates MDSCs to Suppress Tumor Metastasis *via* Reversing Epithelial-Mesenchymal Transition

**DOI:** 10.3389/fonc.2020.00896

**Published:** 2020-06-11

**Authors:** Hao Cheng, Qiming Xu, Xing Lu, Hong Yuan, Tiejun Li, Yuefan Zhang, Xiangshi Tan

**Affiliations:** ^1^Institutes of Biomedical Sciences, Fudan University, Shanghai, China; ^2^Department of Chemistry, Fudan University, Shanghai, China; ^3^Department of Pharmacology, Punan Hospital, Shanghai, China; ^4^Department of Pharmacy, Shanghai General Hospital, Shanghai, China

**Keywords:** cGAMP, myeloid-derived suppressor cells, metastasis, epithelial-mesenchymal transition, STING

## Abstract

The role of cGAMP stimulating cGAS-cGAMP-STING-IRF3 pathway to inhibit tumor growth was well-established. Herein, the efficiency and pharmacological mechanism of cGAMP on regulating tumor metastasis was investigated. The effects of cGAMP regulating CD8^+^ T cells and myeloid-derived suppressor cells (MDSCs) in tumor microenvironment was explored. In this study, we found that cGAMP boosted STING signaling pathway to activate the production of IFN-γ from CD8^+^ T cells, and decreased the population of MDSCs *in vivo*. The metastasis in CT26 tumor bearing mice was inhibited by cGAMP *via* regulating EMT process. cGAMP played an important role in suppressing the production of reactive oxygen species (ROS) and nitric oxide (NO) from MDSCs, abolished the suppressive function of MDSCs to the T cells. All in all, the results indicated that the STING agonist cGAMP activated the production of IFN-γ from CD8^+^ T cells to suppress MDSCs *in vivo*.

## Introduction

Cancer is one of the leading causes of death and a major threat to worldwide public health (1). Tumor metastasis, or the movement of tumor cells from a primary site to colonize distant organs, is the primary cause of death in cancer patients (2). The biological cascade of metastasis includes several discrete steps, which begin with the loss of cellular adhesion, increased tumor cell motility, and invasiveness, tumor cells entering, and surviving in circulation, and finally tumor cells exiting into new tissues that they will eventually colonize to form a new tumor (3, 4). Colorectal and melanoma cancer are the most common malignant tumors, most patients develop metastasis into liver and lung during cancer development (5, 6). This highlights the importance of developing new therapeutic agents to treat metastatic cancer patients.

Epithelial-mesenchymal transition (EMT) is a cell-biological program that confers mesenchymal traits to both normal and neoplastic epithelial cells (7). Chemical small-molecule inhibitors and antibodies directly targeted against EMT-associated factors have been reported to be effective in trials (8). Inhibitors of cell proliferation or angiogenesis, such as erlotinib, gefitinib, and epidermal growth factor receptor (EGFR) inhibitors, could have antitumor effects *via* targeting EMT process (8). Nevertheless, effective broad-spectrum compounds need to be developed to treat tumor metastasis *via* inhibiting EMT. Myeloid-derived suppressor cells (MDSCs) are one of the primary cell populations responsible for regulating the immune system.

The proliferation of MDSCs have a strong ability to inhibit T cell function in tumor-bearing mice (9). MDSCs accumulate during tumor growth and metastasis, and can contribute to cancer cell dissemination *via* regulating EMT (10). The STING signaling pathway is response for the activation of T cells, while T cells can be inhibited by MDSCs in tumor bearing mice.

Cyclic GMP-AMP synthase (cGAS) is a cytosolic DNA sensor that activates innate immune responses by producing the second messenger cGAMP, which activates the adaptor complex protein stimulator of interferon genes (STING/Tmem173). The cGAS-cGAMP-STING-IRF3 pathway mediates the immune defense against infection, detects tumor-derived DNA and generates intrinsic antitumor immunity (11). As an innate immune cytosolic DNA sensor, cGAMP activates the STING signaling pathway to trigger the production of type I interferons (IFNs) and other inflammatory cytokines (12). Also, cGAMP inhibits tumor growth *in vivo*, STING is required for the antitumor effects or acquire sensitive to radiation by activating the STING signaling pathway (13, 14). When tumor-derived DNA is delivered to the cytoplasm of CD8α^+^ dendritic cells (DCs), the STING pathway is activated. Combining cGAMP with irradiation or immune-system checkpoint inhibitors showed synergistic antitumor effects (11). These results indicate that STING signaling plays an important role in tumor suppression. cGAMP has been reported to be an antitumor agent in colon cancer (15); however, the effects of cGAMP in suppressing metastasis were not well-studied. Several cell-based mechanisms are related to tumor metastasis (16), herein we focused on the roles of cGAMP in suppressing metastasis and EMT through Wnt/β-catenin pathway. The Wnt/β-catenin pathway plays a central role in tumor cell proliferation, differentiation, and survival (17).

In this paper, we reported that the STING agonist cGAMP inhibited the liver metastasis of CT26 cells and prevents EMT *in vivo*. cGAMP boosted the STING signaling pathway, which induced the expression of type I interferon (IFNs), and then activated T cells in immune system. This work enhanced our understanding of the molecular mechanism between the STING pathway and metastasis, and promoted the development of an effective immune-based therapy for tumor metastasis.

## Materials and Methods

### Cell Lines and Mice

B16, B16F10, CT26, and MC38 cells were cultured in Dulbecco's modified Eagle's medium (Gibco, Thermo Fisher Scientific, WI, USA) or RPMI-1640 (Gibco, Thermo Fisher Scientific, WI, USA) with 10% fetal bovine serum (Gibco, Thermo Fisher Scientific, WI, USA) at 37°C and 5% CO_2_. Balb/c and C57BL/6 mice were obtained from SIPPR-BK Experimental Animal Co. (Shanghai, China). STING deficiency mice were obtained from the Jackson Laboratory (Bar Harbor, ME, USA). Animal experiments were performed with the approval of institutional guidelines and the local governmental Animal Care Committee of Fudan University.

### Real Time-PCR

Total RNA was extracted using TRIzol reagent (Invitrogen, Carlsbad, CA, USA), and total RNA was used for reverse transcription with Prime Script RT Reagent Kit (Bimake, Shanghai, China) according to the manufacture's protocol. PCR analysis was performed with SYBR Premix Ex-Taq (Bimake, Shanghai, China). The primers were shown in Table S1. Data were normalized to GAPDH expression, and fold changes were calculated by the 2^−ΔΔ*Ct*^ method.

### H&E Staining

Tumor and liver tissue sections were treated and stained as previously reported (15). The tissues were fixed in 4% paraformaldehyde for 24 h, embedded and cut into 5-μm sections, and then stained with H&E reagent. Stained tissue sections were examined under a light microscope (Leica, Wetzlar, Germany).

### Immunohistochemistry and Immunofluorescence

Tumor and liver tissue sections were treated and stained as previously reported (15). The primary antibodies were listed in the Table S2. For immunohistochemistry (IHC) assay, liver tissues were stained with secondary antibody (1:100) for 2 h, and then incubated with SABC and DAB. Nuclei were stained with hematoxylin. For immunofluorescence (IF) staining, incubated with primary antibody (1:100), then followed by Cy™3 AffiniPure goat anti-rabbit IgG secondary antibody (1:200). Nuclei were stained with DAPI (4′,6-diamidino-2-phenylindole). The sections were examined under a microscope (Leica, Wetzlar, Germany).

### Treating Tumor-Bearing and Metastasis Model Mice

To detect the anti-tumor effects of cGAMP, CT26, and B16 cells (2 × 10^5^ cells) were subcutaneously injected into the right flank of the mice. For the metastasis assay, CT26 or MC38 cells (2 × 10^5^ cells) were injected into the spleen of mice which could develop into metastatic nodes in the liver. B16F10 cells (2 × 10^5^ cells) were intravenously injected into the tail of C57BL/6 mice to generate metastatic foci in the lung. Mice were treated with cGAMP daily (20 mg/kg, i.v.) 3 days prior to implantation until the end of the experiments. Luciferase activity was detected using an optical *in vivo* imaging system (IVIS Lumina K; PerkinElmer, Waltham, MA, USA).

### Western Blot

Liver tissues were homogenized in RIPA buffer (Thermo Fisher, Waltham, MA, USA) for protein extraction, and the lysate was centrifuged at 12,000 rpm for 15 min. Protein concentration was measured with the BCA method. The protein samples were separated by SDS-PAGE and transferred to the membranes, then the membranes were incubated with antibodies to detect target proteins.

### Flow Cytometry Analyses

For cytometry assays, we used methods referred to our previous article (15). To generate T cells and MDSCs, tissues were disrupted and filtered through a 40-μm strainer (11). Red blood cells were lysed by ACK lysis buffer. To detect MDSCs, cells were labeled with fluorescence-conjugated antibody CD45, CD11b, Gr-1, Ly6G, and Ly6C, while T cells were labeled for detection with CD3 and CD8. To detect the intracellular cytokine IFN-γ, cells were pretreated with monensin for 4 h, then treated with fixation/permeabilization buffer according to the instructions of the manufacturer, then the cells were stained with IFN-γ antibody. Cells were detected by flow cytometry (BD, CA, USA).

### T Cell Suppression Assay

T cell suppression assay was performed as previously reported. MDSCs were isolated from the spleen in tumor bearing mice with the MDSCs isolation Kit (Miltenyi Biotec, Bergisch Gladbach, Germany) according to the reference. First, single cells harvested from the spleen, and red blood cells were lysed by ACK buffer. Second, single cells were incubated with anti-CD11b and anti-Gr-1 antibodies. Third, cells were incubated with beads. Then the cells were isolated *via* the magnetic sorting.

T cells were enriched from the spleens with the Mouse T Cell Enrichment Kit (ebioscience, San Diego, CA, USA). T cells were labeled with CFSE (carboxyfluorescein succinimidyl ester) (1 mol/L) for 15 min at 37°C. T cells co-cultured with MDSCs for 72 h in RPMI-1640 supplemented with 10% FBS which pre-coated with anti-CD3 (1 μg/mL) and anti-CD28 (2 μg/mL) antibody. The proliferation of T cells was detected with CFSE *via* flow cytometry.

### ROS and NO Detection

Single-cell suspensions were prepared from spleens as previously described (15). MDSCs were harvested by the MDSCs isolation Kit (Miltenyi Biotec, Bergisch Gladbach, Germany) as it indicated before. MDSCs were seeded into the 6 well plates and treated with cGAMP at 50 and 10 μg/mL for 24 h, respectively. The production of ROS (reactive oxygen species) and NO (nitric oxide) were measured by labeling DCFH-DA (2′,7′-dichlorodihydrofluorescein diacetate) and DAF-FM DA (3-Amino-4-aminomethyl-2′,7′-difluorescenin, diacetate) probes, respectively (Beyotime, Shanghai, China) in MDSCs, then detected by flow cytometry.

### Statistical Analysis

All data are from at least three independent experiments and are presented as mean ± *SD*. Two-tailed Student's *t*-tests were used to test the differences, with ^*^P < 0.05, ^**^P < 0.01, ^***^P < 0.001, and ^##^*P* < 0.01 considered statistically significant.

## Results

### STING Deficiency Increases Tumor Growth and Metastasis, and Reduces Immune Cell Infiltration in Tumor-Bearing Mice

**Figure 1:**
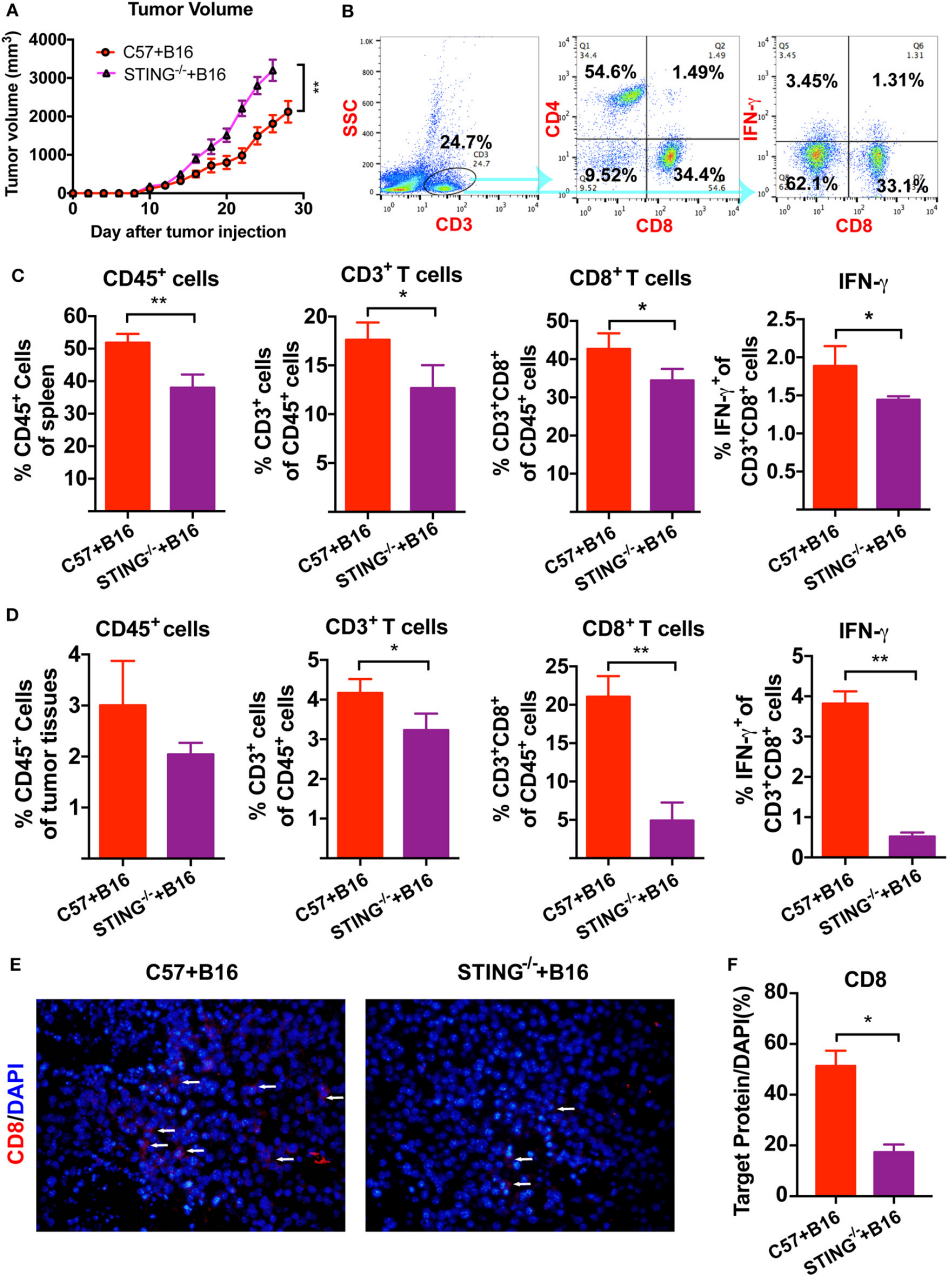
STING deficiency increases the growth of B16 tumors and reduces infiltrating immune cells. C57 and STING^−/−^ mice were injected with B16 cells. **(A)** Tumor volumes of B16 tumor bearing mice were detected in C57 and STING deficiency mice. **(B)** T cells (CD3^+^ cells and CD3^+^CD8^+^ cells) and IFN-γ released from CD3^+^CD8^+^ cells were detected by flow cytometry. **(C)** The quantification of CD45^+^ cells, T cells, and IFN-γ were showed in spleen. **(D)** The quantification of CD45^+^ cells, T cells, and IFN-γ were showed in tumor tissues. **(E)** The expression of CD8 was detected in tumor tissues as shown by immunofluorescence. **(F)** The expression level of CD8 in **(E)** was showed. The values are expressed as mean ± *SD*. (*n* = 6; *P < 0.05 **P < 0.01, vs. the control group).

We have previously reported that cGAMP boosted the immune system to induce DCs and T cells activation, which increased antitumor activity in tumor-bearing mice. Here, B16 melanoma cells were injected into the right flank of the C57BL/6J and STING^−/−^ mice. Compared with the control group, tumor volumes in STING^−/−^ B16 tumor bearing mice increased (Figure 1A). Furthermore, more tumor metastatic nodes were found in the lungs of STING^−/−^ B16F10 tumor-bearing mice, compared with the C57BL/6J controls (Figure S1). Then, T cells and IFN-γ were detected by flow cytometry in spleens and tumor tissues (Figure 1B). Compared with the C57BL/6J mice, the numbers of immune cells decreased in STING^−/−^ mice, including CD45^+^ leukocytes, CD3^+^ T cells and CD3^+^CD8^+^ T cells. The expression of IFN-γ released from CD3^+^CD8^+^ T cells was also decreased in spleen significantly (Figure 1C). At the same time, the immune cells in tumor tissues showed similar results regarding the detection of immune cells and IFN-γ (Figure 1D). Compared with the spleen, the number of T cells decreased rapidly in tumor tissues, especially for the CD3^+^CD8^+^ T cells and IFN-γ. Furthermore, the expression level of CD8 in tumor tissues were detected by IF (Figure 1E), which showed significant decreasing expression in STING^−/−^ tumor bearing mice (Figure 1F). The arrow showed the positive expression of the target protein in the tumor tissues. These results suggested that STING deficiency significantly altered the immune system, causing T cell dysfunction.

### CD11b^+^Gr-1^+^ Cells Are Significantly Increased in the STING^−/−^ Tumor Mice

**Figure 2:**
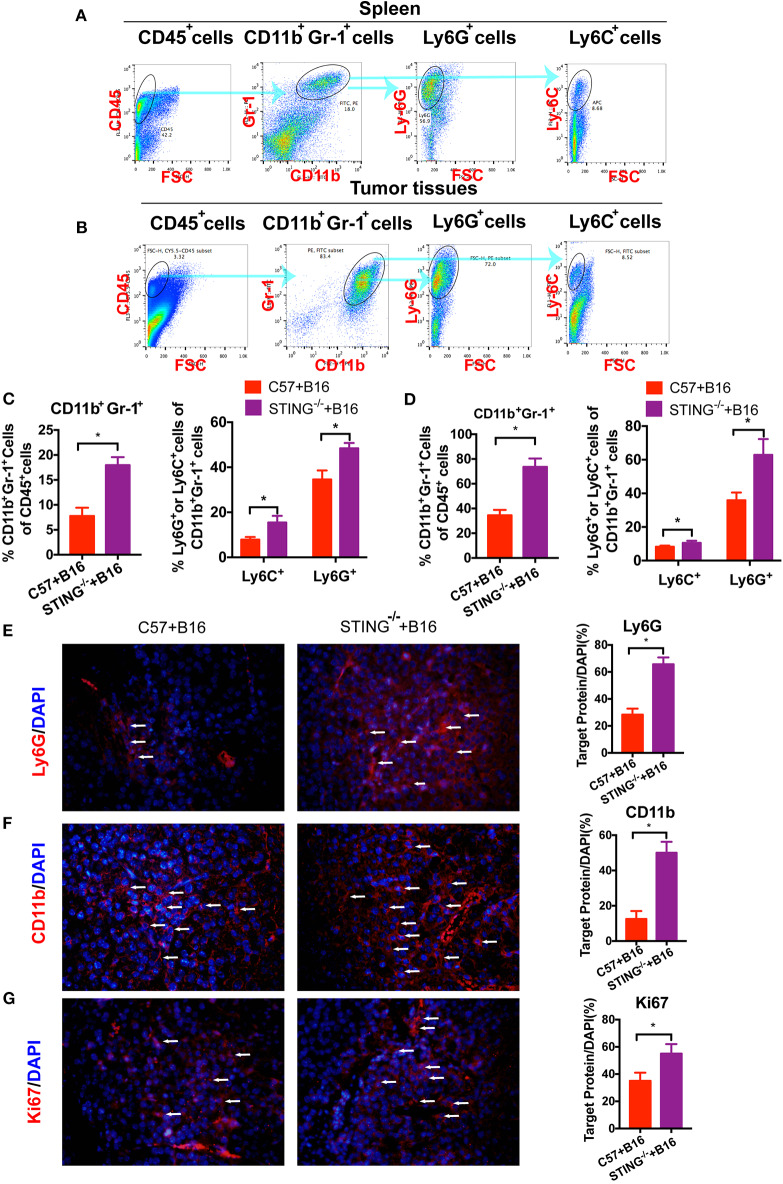
CD11b^+^Gr-1^+^ MDSCs are significantly increased in STING-deficient mice. C57 and STING^−/−^ mice were injected with B16 cells. **(A)** The population of MDSCs in spleens was detected by flow cytometry. **(B)** The population of MDSCs in tumor tissues was detected by flow cytometry. **(C)** Quantification of the population of MDSCs, PMN-MDSCs and M-MDSCs in spleens. **(D)** Quantification of the population of MDSCs, PMN-MDSCs, and M-MDSCs in tumor tissues. **(E,F)** Representative images of the PMN-MDSCs markers, CD11b, and Ly6G, detected by IF in tumor tissues. **(G)** Ki67 was detected by IF to indicate the proliferation of tumor cells. The values are expressed as mean ± *SD*. (*n* = 6; *P < 0.05 vs. the control group).

As reported, T cells and DCs function was abolished or suppressed in STING^−/−^tumor bearing mice (15). Hence, the immune suppressive cells were detected *in vivo*, such as MDSCs. To investigate the function of MDSCs in STING^−/−^ mice, we classified MDSCs as CD11b^+^Ly6G^+^Ly6C^−^ (polymorphonuclear (PMN)-MDSCs) and CD11b^+^Ly6G^−^Ly6C^+^ (monocytic (M)-MDSCs). At day 20, the population of MDSCs in spleens (Figure 2A) and tumors (Figure 2B) were detected by flow cytometry. The population of total MDSCs increased highly in both spleens (Figure 2C) and tumor tissues (Figure 2D) with the deficiency of STING. As PMN-MDSCs comprised major population of MDSCs *in vivo*, PMN-MDSCs increased rapidly in STING^−/−^ mice, as well as the M-MDSCs (Figure 2C). In STING^−/−^ mice (Figure 2D), total MDSCs and PMN-MDSCs increased ~40% and 26% in tumor tissues, respectively. Also, the population of M-MDSCs increased in STING^−/−^ tumor bearing mice significantly. The abundance of PMN-MDSCs in tumor tissues was further confirmed by IF with CD11b and Ly6G (Figures 2E,F), it showed significant increasing in STING^−/−^ mice. Cell proliferation was significantly increased in STING^−/−^ mice, which was confirmed by Ki-67 staining (Figure 2G). Thus, this is a new way to study STING function.

### cGAMP Inhibits Tumor Growth and Metastasis

**Figure 3:**
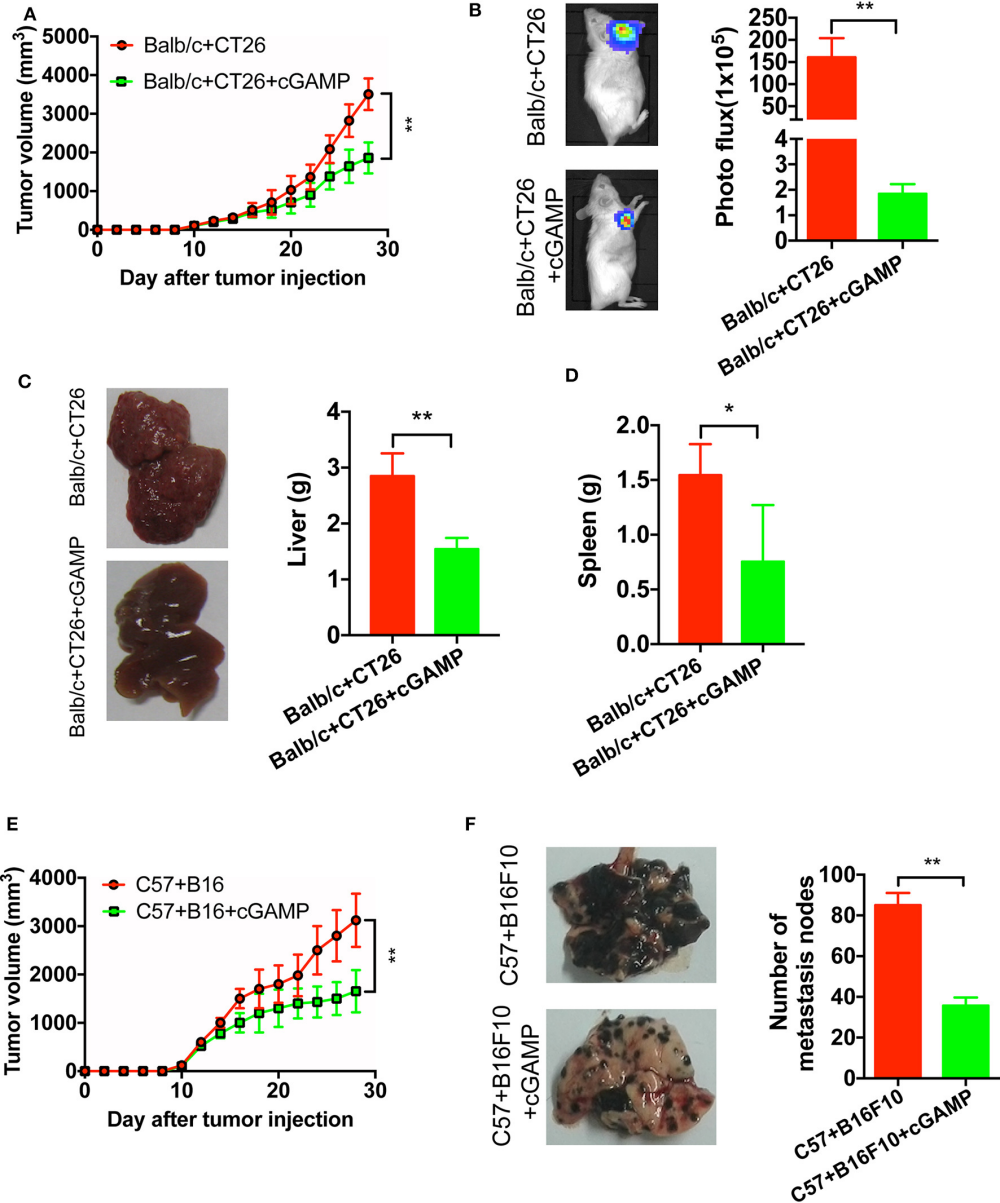
cGAMP inhibits tumor growth and metastasis *in vivo*. Mice were treated with cGAMP (20 mg/kg, i.v.) daily until the end of the experiments, 3 d prior to injecting tumor cells. CT26 or B16 cells were injected into the right flank of the mice, while for the metastasis model, CT26 cells were injected into the spleen of mice and B16F10 cells were injected into the tail vein. Mice were sacrificed and harvested the main organs at day 28. **(A)** cGAMP decreased the tumor volumes of CT26 tumor-bearing mice. **(B)** CT26-luc cells were used to measure tumor volumes in tumor-bearing mice; cGAMP decreased the radiance of CT26-luc mice. **(C)** cGAMP decreased the metastasis of CT26 tumor cells in liver tissues. **(D)** The weight of spleen was detected as shown. **(E)** cGAMP decreased the tumor volumes of B16 tumor-bearing mice. **(F)** Lung tissues of B16F10 metastasis tumor mice are shown; cGAMP significantly decreased the metastatic nodes in tumor bearing mice. The values are expressed as mean ± *SD*. (*n* = 6; *P < 0.05 **P < 0.01 vs. the control group).

It has demonstrated that cGAMP boosts the innate immune system *via* stimulating STING signal to exhibit antitumor effects in colon cancer model (15). To develop xenograft tumor model mice, B16, and CT26 cells were cultured and subcutaneously injected into the right flank of the C57 or Balb/c mice. It showed that cGAMP significantly inhibited the growth of CT26 tumors in *vivo* (Figures 3A,B). Meanwhile, CT26 cells were injected into the spleen of Balb/c mice to establish the liver metastasis model. At day 26th, mice were sacrificed and harvested tumor tissues and livers. Liver weight decreased in cGAMP-treated mice (1.54 ± 0.20 g) compared with the untreated group (2.84 ± 0.41 g), which indicated that cGAMP effectively inhibited the formation and growth of metastatic liver nodules (Figure 3C). Additionally, the average spleen weight decreased in cGAMP-treated mice (0.75 ± 0.51 g) compared with that of the untreated control group (1.54 ± 0.28 g) at day 26th (Figure 3D). Thus, cGAMP suppressed the metastasis of CT26 tumor cells *in vivo*.

We also determined the effects of cGAMP on melanoma cancer. With the treatment of cGAMP, the tumor volume in B16 tumor bearing mice decreased significantly at day 26th (Figure 3E). After mice injected with B16F10 cells, we observed these metastatic nodes decreased in the lungs of the cGAMP-treated mice at day 26th (Figure 3F). These results suggested that cGAMP suppressed tumor growth and metastasis of CT26, B16, and B16F10 cells *in vivo*.

### cGAMP Inhibits Tumor Growth and Metastasis by Suppressing MDSCs

**Figure 4:**
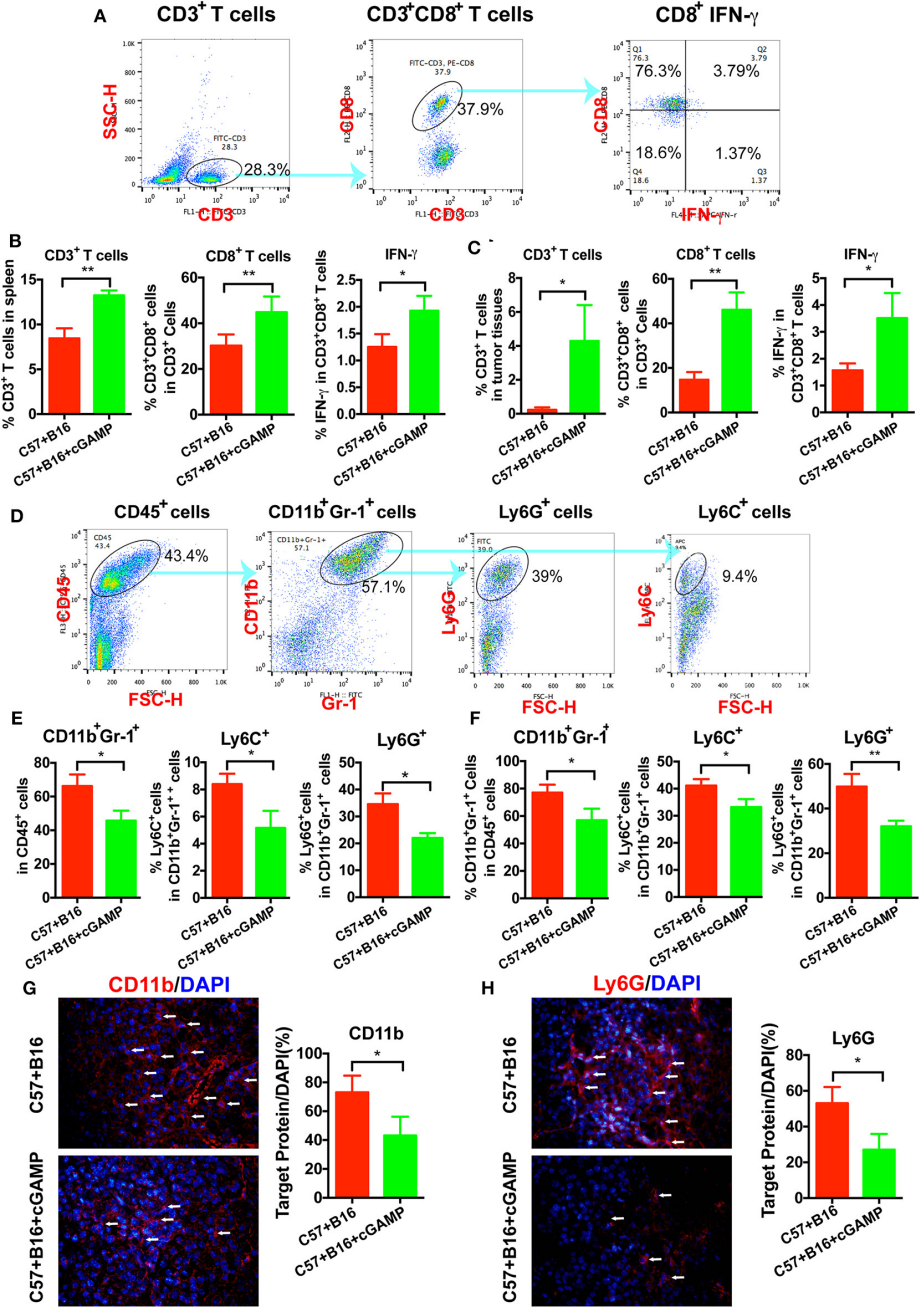
cGAMP inhibits tumor growth and metastasis *via* regulating MDSCs *in vivo*. Mice were treated with cGAMP (20 mg/kg, i.v.) daily 3 d before B16 cells were injected into their right flank. **(A)** CD3^+^ T cells, CD3^+^CD8^+^ T cells, and IFN-γ released from CD3^+^CD8^+^ T cells were detected by flow cytometry. **(B)** cGAMP increased the population of CD3^+^ T cells, CD3^+^CD8^+^ T cells, and IFN-γ in the spleen. **(C)** cGAMP increased the population of CD3^+^ T cells, CD3^+^CD8^+^ T cells, and IFN-γ in the tumor tissues. **(D)** Diagram of the flow cytometry analyses of MDSCs (18). The population of total MDSCs and its sub-population in the spleens **(E)** and tumor tissues **(F)** were detected by flow cytometry. **(G,H)** The expression of CD11b and Ly6G were detected by IF in tumor tissues. The values are expressed as mean ± *SD*. (*n* = 6; *P < 0.05 **P < 0.01 vs. the control group).

To determine whether CD8^+^ T cells could be triggered by cGAMP *in vivo*, C57 mice were inoculated with B16 cells and treated with cGAMP or saline daily. Mice were euthanized on day 7, as described before (15), and splenic CD3^+^CD8^+^ T cells were detected by flow cytometry (Figure 4A). The percentage of CD3^+^CD8^+^ T cells and IFN-γ in spleen tissues were increased after cGAMP treatments (Figure 4B). Meanwhile, T cells were activated in tumor tissues with the detection by flow cytometry (Figure 4C). It indicated that cGAMP activated T cells by boosting the innate immune system *in vivo*.

To verify whether cGAMP played a role in regulating MDSCs to exhibit antitumor effects, we detected MDSCs by flow cytometry *in vivo* (Figure 4D). It showed that total MDSCs in spleens decreased with the treatment of cGAMP (Figure 4E), which indicated that cGAMP inhibited the proliferation of MDSCs. For further investigation, PMN-MDSCs and M-MDSCs were analyzed. Both the PMN-MDSCs and M-MDSCs decreased after cGAMP treatment (Figure 4E). Also, we found that the population of MDSCs, PMN-MDSCs, and M-MDSCs decreased in tumor tissues following cGAMP administration (Figure 4F). We determined whether cGAMP could regulate other immune cells *in vivo*.

We also examined the expression of MDSCs markers by IF. As it shown, CD11b (Figure 4G) and Ly6G (Figure 4H) were down-regulated in cGAMP-treated group which indicated lower MDSCs presentation in tumor tissues. These data indicated that cGAMP inhibited tumor growth and metastasis by regulating MDSCs *in vivo*.

### cGAMP Depresses the Suppressive Effects of MDSCs to T Cells

**Figure 5:**
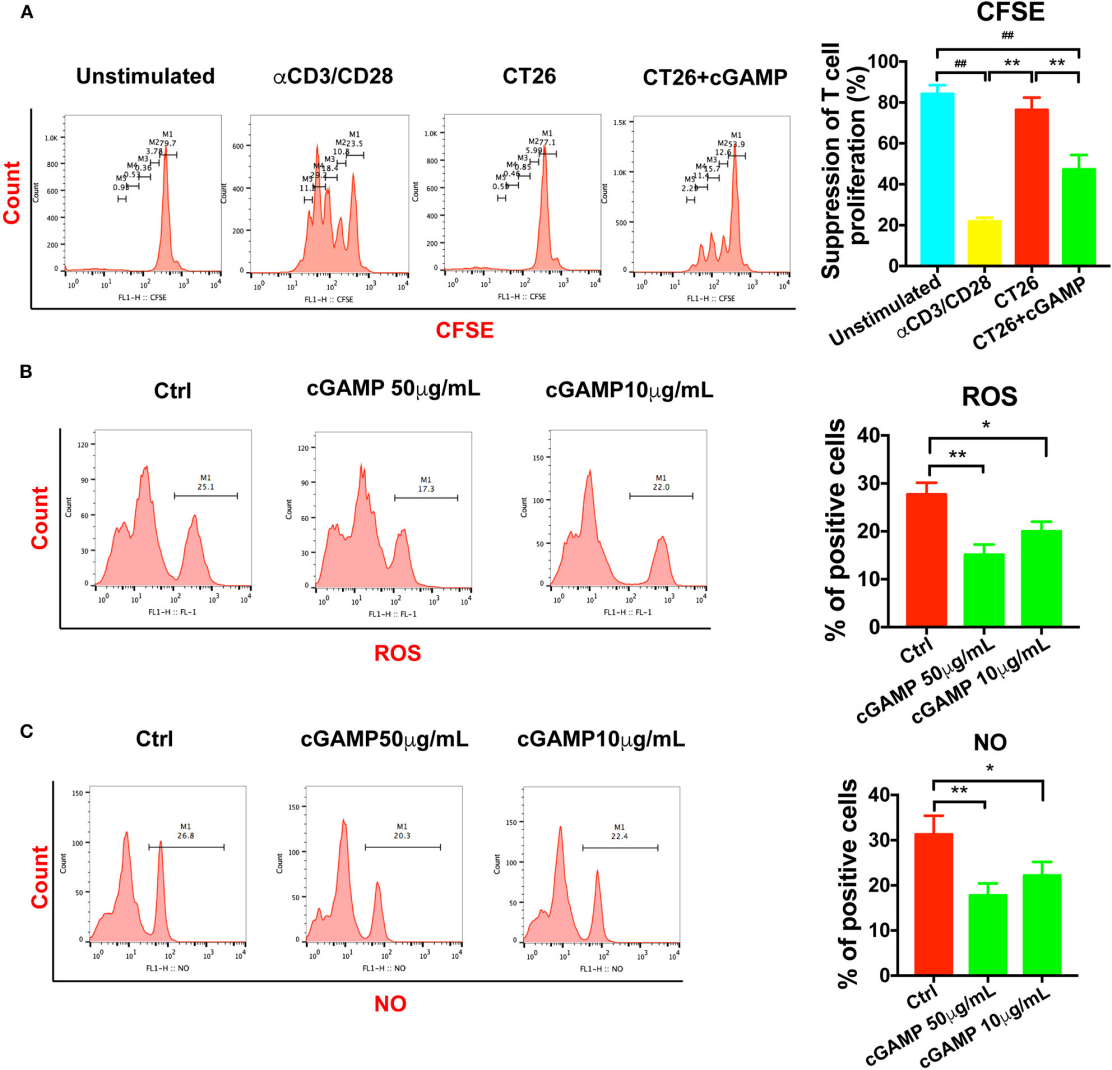
cGAMP delayed the suppression effects of MDSCs *in vitro*. MDSCs and T cells were isolated with the Kit. **(A)** T cell suppression assay was performed to detect the immune suppression effects of MDSCs in each group. The ratio of T cells: MDSCs were 2:1. Representative data of three independent experiments are shown as mean ± *SD*. (*n* = 3; **P < 0.01 vs. the CT26 group; ^**##**^*P* < 0.01 vs. the negative control group). **(B)** The concentration of ROS was determined with the flow cytometry. **(C)** The concentration of NO was determined with the flow cytometry. Representative data of three independent experiments are shown as mean ± *SD*. (*n* = 3; *P < 0.05, **P < 0.01 vs. the control group).

It is well-established that MDSCs have immune suppressive functions through the production of NO, Arg-1, and ROS. In order to determine whether cGAMP could inhibit the suppressive function of MDSCs, we detected T cell proliferation *in vitro*. As shown in Figure 5A, T cell proliferation was assessed by CFSE labeling. MDSCs were isolated from tumor bearing mice, which showed strong immune suppressive effects to T cells, while MDSCs in cGAMP treated mice showed decreased immune suppression effects (Figure 5A). Meanwhile, NO and ROS could suppress T cell function in tumor bearing mice to support tumor growth. Consistent with the decreased suppressive effects of MDSCs by cGAMP, the concentration of ROS (Figure 5B) and NO (Figure 5C) decreased with the treatment of cGAMP *in vitro*. The decreasing production of ROS and NO explains the reason why cGAMP could inhibit MDSCs. All the results showed that cGAMP could decrease the immune suppressive effects to T cells *via* reducing the concentration of ROS and NO.

### STING Deficiency Increases Metastasis of MC38 Colon Cancer Cells

**Figure 6:**
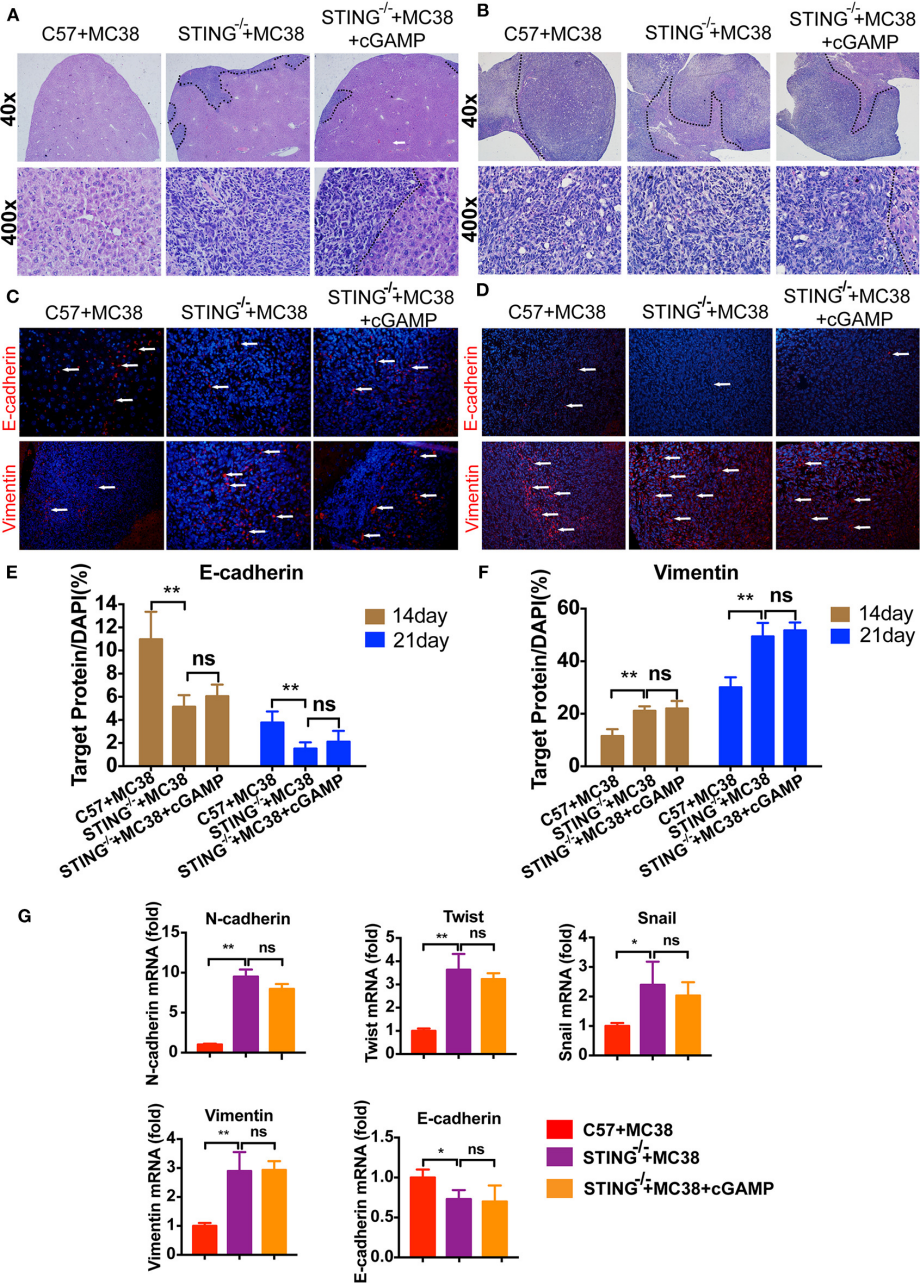
STING deficiency increased the metastasis of MC38 colon cancer cells. Mice were treated with cGAMP (20 mg/kg, i.v.) daily 3 d before MC38 cells were injected into their spleens. **(A)** H&E staining of liver tissues to detect metastatic MC38 cancer cells on days 14, respectively. **(B)** H&E staining of liver tissues to detect metastatic MC38 cancer cells on days 22. **(C)** IF staining of liver tissues for E-cadherin and Vimentin on day 14. **(D)** IF staining of liver tissues for E-cadherin and Vimentin on day 22. **(E)** The quantification of E-cadherin was measured as shown. **(F)** The quantification of Vimentin was measured as shown. **(G)** RT-PCR analysis of N-cadherin, Twist, Snail, Vimentin, and E-cadherin. Representative data of three independent experiments are shown as mean ± *SD*; (*n* = 6; *P < 0.05, **P < 0.01 vs. the control group).

It has reported that STING deficiency promotes tumor growth in mice, but whether STING regulates metastasis is unknown. Thus, we injected MC38 cells into the spleen of wild-type (WT) and STING^−/−^ mice to establish liver metastases. On days 14 and 22, the mice were sacrificed, and livers were fixed and embedded in paraffin, stained with H&E. H&E staining showed that liver metastases occurred in STING^−/−^ mice on day 14 (Figure 6A), while no metastases had formed in the WT group, the tumor metastasis appeared in cGAMP treated group (Figure 6A). On day 22, both the WT and STING^−/−^ mice showed metastatic nodules in the liver, and cGAMP had no effects in STING^−/−^ mice (Figure 6B). The results indicated that cGAMP had no direct anti-metastatic activity in STING^−/−^ mice (Figures 6A,B). To examine whether the EMT inhibitory effects of cGAMP were STING-dependent, STING^−/−^ mice with liver metastases were treated with cGAMP daily. The EMT markers were detected by IF in tumor tissues at day 14 (Figure 6C) and day 22 (Figure 6D), respectively. At day 14 and 21, the expression of E-cadherin and Vimentin didn't change a lot in two groups. cGAMP had no effects in regulating the expression of E-cadherin and Vimentin (Figures 6E,F). Thus, cGAMP seemed to have no effects in STING^−/−^ mice in regulating EMT *in vivo*. The mRNA expression levels of EMT markers were quantified by RT-PCR (Figure 6G).

Furthermore, the expression of cytokines was also examined by RT-PCR (Figure S2). Compared with the WT group, STING^−/−^ mice showed decreased expression of TNF-α, IL-2, IFN-γ, and IFN-β, similar to previous reports (15). Together, these results confirmed that the STING pathway is critical for the liver metastasis of colon cancer.

### cGAMP Regulates EMT-Related Genes *Via* the Wnt/β-Catenin Pathway to Inhibit Metastasis

**Figure 7:**
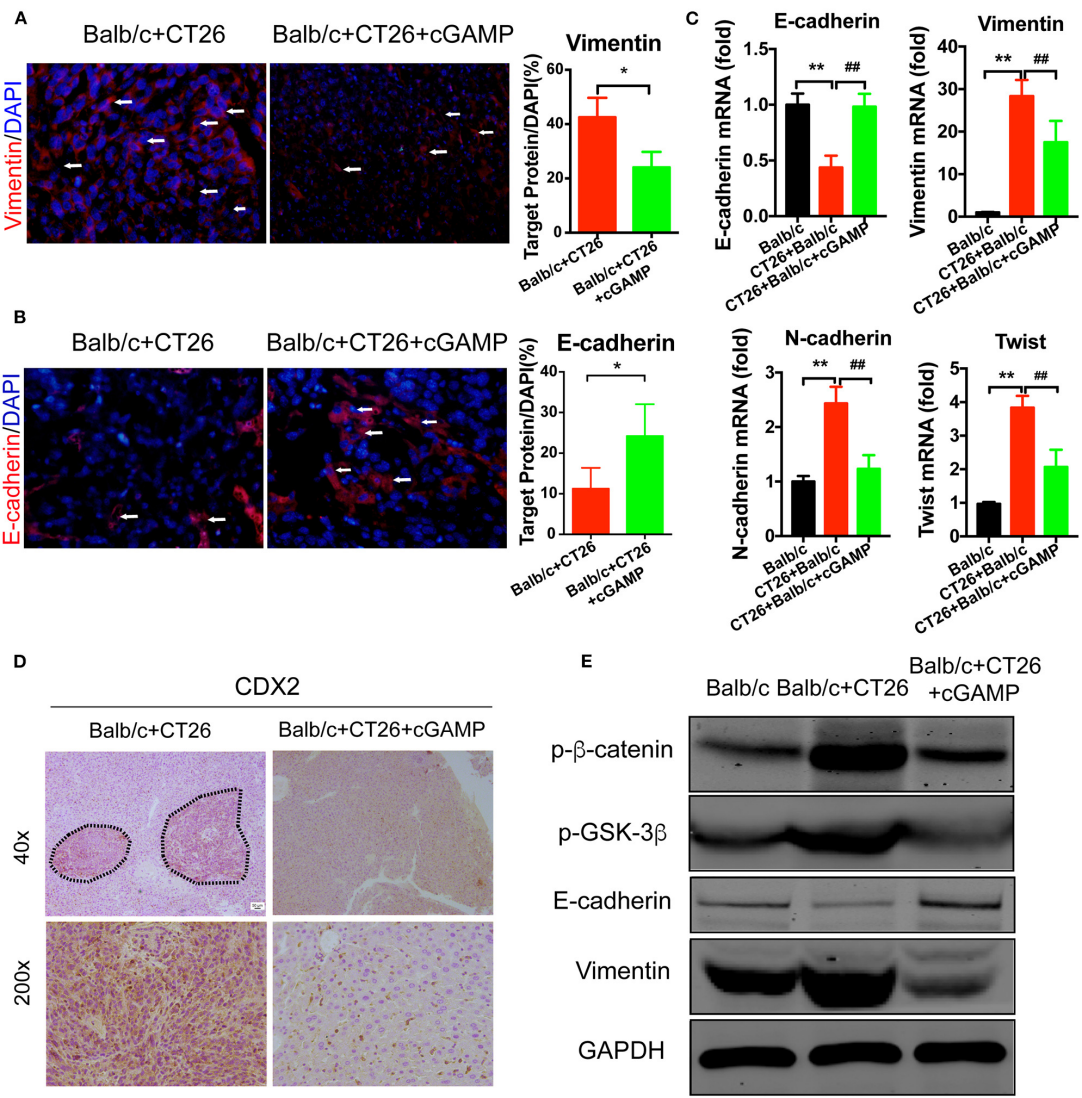
cGAMP inhibits tumor metastasis by regulating EMT-related genes in the Wnt/β-catenin signaling pathway *in vivo*. Mice were treated with cGAMP (20 mg/kg, i.v.) daily, 3 d before CT26 cells were injected into their spleens. **(A,B)** IF staining for Vimentin and E-cadherin in liver tissues. **(C)** RT-PCR analysis of E-cadherin, Vimentin, N-cadherin, and Twist expression in liver tissues of CT26 tumor bearing mice. **(D)** CDX2 expression was analyzed by immunohistochemistry. **(E)** Western blot analysis of Wnt-β/catenin signal pathway in liver tissues. The values are expressed as mean ± *SD*. (*n* = 6; *P < 0.05 **P < 0.01, and ^*##*^*P* < 0.01, vs. the control group).

To investigate whether cGAMP played a role in suppressing metastasis *via* EMT, EMT markers were examined by IF and RT-PCR (Figures 7A–C). These results indicated that cGAMP increased the expression of epithelial markers and down-regulated the expression of mesenchymal markers in liver tissues during EMT. Furthermore, CDX2, which is the marker of colon cancer cells, were down regulated in cGAMP treated group by immunohistochemistry staining (Figure 7D). The anti-tumor cytokines were up-regulated in cGAMP treatment (Figure S3), which was similar to what we previously reported (15). Later, we examined the mechanism of cGAMP on Wnt/β-catenin pathway by western blot. These data indicated that cGAMP treatment decreased the expression of p-β-catenin and p-GSK-3β. These results strongly suggested that cGAMP blocked the phosphorylation of GSK-3β and the accumulation of β-catenin (Figure 7E). Overall, these results confirmed the suppressive effects of cGAMP on Wnt/β-catenin signaling during tumor metastasis.

## Discussion

Recent advances in the study of innate immune system have recognized the importance of immunotherapy, and immunotherapy has become one of the key strategies for cancer treatment. The STING innate immunity pathway played a pivotal role in antiviral defense, and STING activator cGAMP possesses significant antitumor activity in mice by directly stimulating this pathway (15). Furthermore, cGAMP augments the antitumor activity of 5-FU, radiation, and CD47 antibody, reducing the toxicity of 5-FU (15, 19, 20). We previously found that cGAMP boosted the STING signal pathway to inhibit tumor growth (15). The roles of STING signal pathway and its agonist have been demonstrated in antitumor activity, and the efficiency, and pharmacological mechanism of cGAMP to suppress tumor metastasis was confirmed in our study.

Tumor microenvironment contains numerous cell types, including cancer cells, lymphocytes, blood vessels, bone marrow-derived inflammatory cells, adipose cells, and fibroblastic cells (21). Adipocytes play a key role in tumor microenvironment. The adipose cells could secret adipokines, growth factor, and pro-inflammatory cytokines, which could support tumor growth and metastasis (22). In mice, the bone marrow-derived cells, MDSCs are characterized by the differentiation markers CD11b and Gr-1 (23). It is well-established that MDSCs consist of two subtypes: PMN-MDSCs and M-MDSCs, while the PMN-MDSCs possess majority of MDSCs in tumor-bearing mice (23). The roles of MDSCs in suppressing tumor growth by enhancing tumor survival, angiogenesis, metastasis and the formation of pre-metastatic niches have been previously confirmed (24). Immune suppression is the main feature of MDSCs, while the M-MDSCs are more suppressive than PMN-MDSCs (25). MDSCs have various mechanisms to suppress T-cell function, including generating high levels of NO, ROS, and arginase-1 *in vivo* (26). The suppressive function of MDSCs to T cells was reduced after cGAMP treatment. In our study, we demonstrated that cGAMP delayed immune suppressive function of MDSCs to CD8^+^ T cells. It had reported that drug resistance is regulated by the CCR2^+^ M-MDSCs, and the depletion of CCR2 delays the immunosuppressive function of MDSCs (27). The CCR2^+^ MDSCs accumulated in obese tumor bearing mice through the production of CCL-2 (28). Zhang et al. reported that STING plays a vital role in regulation of MDSCs differentiation and anti-tumor immunity in Epstein-Barr virus (EBV)-associated nasopharyngeal (NPC) (29). MDSCs accumulated in the adipose tissue due to the chronic inflammation in the tumor tissues. The level of IL-6, TNF-α, and other pro-inflammatory mediators were elevated in obesity people. Theses mediators could induce the accumulation and differentiation of MDSCs in tumor microenvironment (30). The percentage of M-MDSCs and PMN-MDSCs is negatively related with the therapy effects of anti-PD1 and anti-CTLA-4 (31). Also, we provided the first evidence that STING deficiency increased the proliferation of MDSCs, including the M-MDSCs and PMN-MDSCs. Hence, the elimination of cGAMP to the MDSCs played a vital role in clinical tests. Also, the activation of STING pathway become the approach to adjust the obesity-induced inflammation and metabolic diseases (32). This provides us another evidence that the STING pathway could regulate inflammation in adipose tissues, which decrease the accumulation of MDSCs *in vivo*. When the MDSCs differentiate into tumor associated macrophages (TAMs) or DCs, it could reduce the population and suppressive function of MDSCs (33). But, whether cGAMP regulates MDSCs differentiation remains further investigation. Mechanism between the increasing type I IFNs and decreasing MDSCs need further investigation. Also, whether cGAMP could regulate target proteins on MDSCs such as MMPs and STAT3 remains unknown.

Metastasis occurs when the tumor cells adapt to a new tissue microenvironment escape from the primary tumor (34). Adipose tissue and adipocytes support tumorigenesis and metastasis (35). Theoretically, several steps are analyzed in metastasis assays, including tumor cells invasion, survival in the bloodstream and metastatic colonization (36). One of the most intensively studied molecular mechanisms of metastasis is epithelial-mesenchymal transition (EMT), EMT is a critical step in tumor dissemination and metastasis (37). EMT can adversely promote carcinoma progression through a variety of mechanisms (8). The changes in gene expression that inhibit an epithelial phenotype and activate the mesenchymal phenotype are thus the main regulators of this process (16). The down-regulation of E-cadherin is a hallmark of EMT (16). The M-MDSCs and PMN-MDSCs infiltrate in the primary tumor and distant, and the recruitment of bone MDSCs from metastatic lungs promotes the proliferation of tumor cells to enhance metastasis (38). It reported that primary tumors attracted a subset of immune cells that promoted the motility, dissemination and metastasis of cancer cells. It was also found that CXCL5 attracted MDSCs to the primary tumor site to induce EMT *in vivo* (39). PMN-MDSCs infiltrate tumor tissues to support metastatic growth by reverting EMT/cancer stem cell phenotypes and promoting tumor cell proliferation (10). Also, it has reported that CXCR2^+^ MDSCs promote breast cancer growth and metastasis *via* inducing cancer cell EMT thus promoting T cells exhaustion (40). Our results showed that STING signal pathway plays an important role in inhibiting the proliferation of MDSCs and reverting the EMT process to delay tumor metastasis *in vivo*.

The contact between MDSCs and tumor cells enhance the expression of COX-2, which activate the β-catenin pathway, resulted in EMT (41). The Wnt/β-catenin pathway could regulate tumor growth, differentiation, invasion and metastasis. The activation of Wnt/β-catenin signaling could promote the metastasis of breast cancer (42), while the suppression of Wnt signaling resulted in the suppression of EMT and tumor metastasis. It has proved that type I IFNs and Wnt signaling converge at GSK-3β-TBK1 binding (43), and inhibiting Wnt signaling by miR-34(g) resulted in enhanced signaling through the IRF3-STING pathway, promoting an antiviral state (43). STING deficiency showed the abortion of type I IFNs, decreasing of DCs and CD8^+^ T cells. It had proved that the activation of STING signal pathway in T cell induced the death of T cell *in vitro* while had no cytotoxic *in vivo* (44). One of the major reasons is that the ENPP1 (Ecto-nucleotide pyrophosphatase phosphodiesterase 1), which is a type II transmembrane glycoprotein, could hydrolyze 2′3′-cGAMP and regulate cGAS-STING pathway in innate immune system (45). Our results showed that STING signal pathway plays an important role in inhibiting the proliferation of MDSCs and reverting EMT process to delay tumor metastasis *in vivo*.

In summary, the metastasis of CT26 cancer cells was significantly suppressed by the stimulation of the STING pathway. It boosted the innate immune system to activate CD3^+^CD8^+^ T cells and related cytokines. Also, cGAMP inhibited tumor metastasis *via* regulating EMT through the Wnt/β-catenin pathway. cGAMP inhibited the liver metastasis of colorectal cancer cells by suppressing EMT. Taken together, our results suggest that cGAMP is a novel immune adjuvant that activates the STING signaling pathway to inhibit tumor metastasis.

## Data Availability Statement

All datasets generated for this study are included in the article/Supplementary Material.

## Ethics Statement

The animal study was reviewed and approved by Fudan University.

## Author Contributions

HC performed most of the experiment and wrote the manuscript. XT designed the experiment and revised the manuscript. QX, XL, and HY participated in the animal experiment. TL and YZ participated in organizing the figures.

## Conflict of Interest

The authors declare that the research was conducted in the absence of any commercial or financial relationships that could be construed as a potential conflict of interest.
