# Supplementary material for: Activation of STING by cGAMP Regulates MDSCs to Suppress Tumor Metastasis via Reversing Epithelial-Mesenchymal Transition
Source: Front Oncol. 2020 Jun 11;10:896. doi: 10.3389/fonc.2020.00896 (PMC7300176; doi:10.3389/fonc.2020.00896)
Supplement: Supplementary file 1 [file Data_Sheet_1.docx]

**Supplementary Material**

**Abbreviations:** cGAS: Cyclic GMP-AMP synthase; cGAMP: Cyclic AMP-GMP; CDX2: Caudal Type Homeobox 2; CFSE: carboxyfluorescein succinimidyl ester; CTLA-4: Cytotoxic T-lymphocyte associated protein 4; CXCL5: C-X-C motif chemokine ligand 5; DAF-FM DA: 3-Amino-4-aminomethyl-2’,7’-difluorescenin, diacetate; DAPI: 4’,6-diamidino-2-phenylindole; DCFH-DA: 2’,7’-dichlorodihydrofluorescein diacetate; DCs: dendritic cells; EGFR: epidermal growth factor receptor; EMT: epithelial-mesenchymal transition; GSK-3β: glycogen synthase kinase 3β; IFNs: interferons; IHC: immunohistochemistry; IF: immunofluorescence; IRF3: Interferon regulatory Factor 3; MDSC: myeloid-derived suppressor cells; M-MDSCs: monocytic MDSCs; PMN-MDSCs: polymorphonuclear MDSCs; NO: nitric oxide; PD1: Programmed cell death protein 1; ROS: reactive oxygen species; SDS-PAGE: sodium dodecyl sulfate-polyacrylamide gel electrophoresis; STING: stimulator of interferon genes protein; TAMs: tumor associated macrophages

**Table S1:** Primer sequences:

| Gene Name | Forward/ Reverse | Primer Sequence |
| --- | --- | --- |
| GAPDH | Forward | 5′-CCAGCCCAGCAAGGATACTG-3′ |
|  | Reverse | 5′-GGTATTCGAGAGAAGGGAGGGC-3′ |
| IFN-β | Forward | 5′-TCCGAGCAGAGATCTTCAGGAA-3′ |
|  | Reverse | 5′-TGCAACCACCACTCATTCTGAG-3′ |
| IFN-γ | Forward | 5′-AGCAACAGCAAGGCGAAAA-3′ |
|  | Reverse | 5′-CTGGACCTGTGGGTTGTTGA-3′ |
| IL-2 | Forward | 5′-TCAGCAACTGTGGTGGACTT-3′ |
|  | Reverse | 5′-ACATTTTTGAGCCCTTGGGG-3′ |
| N-cadherin | Forward | 5′-ACAGTGGCCACCTACAAAGG-3′ |
|  | Reverse | 5′-CCGAGATGGGGTTGATAATG-3′ |
| Twist | Forward | 5′-GACGAGCTGGACTCCAAGAT-3′ |
|  | Reverse | 5′-CCATCCTCCAGACCGAGAAG-3′ |
| Snail | Forward | 5′-CCTCCCTGTCAGATGAGGAC-3′ |
|  | Reverse | 5′-CCAGGCTGAGGTATTCCTTG-3′ |
| Vimentin | Forward | 5′-GAGAACTTTGCCGTTGAAGC-3′ |
|  | Reverse | 5′-GCTTCCTGTAGGTGGCAATC-3′ |
| E-cadherin | Forward | 5′-TTGCACCGGTCGACAAAGGAC-3′ |
|  | Reverse | 5′-TGGATTCCAGAAACGGAGGCC-3′ |

**Table S2**: Antibodies list:

| Antibody | Source of primary mAb |
| --- | --- |
| CDX2 | Cell Signaling Technology, Danvers, MA, USA |
| E-cadherin | Cell Signaling Technology, Danvers, MA, USA |
| Vimentin | Cell Signaling Technology, Danvers, MA, USA |
| Ki-67 | Abcam, Cambridge, UK |
| CD8 | Abcam, Cambridge, UK |
| CD11b | Abcam, Cambridge, UK |
| Ly6G | Abcam, Cambridge, UK |

**Figure S1: The metastatic foci in the lung tissues.**

The B16F10 cells (2 × 10^5^ cells) were intravenously injected into the tail of C57BL/6J and STING deficiency mice to generate metastatic foci in the lung. The tumor metastasis nodes of B16F10 mice were measured and shown as follow. (^**^*P*<0.01).


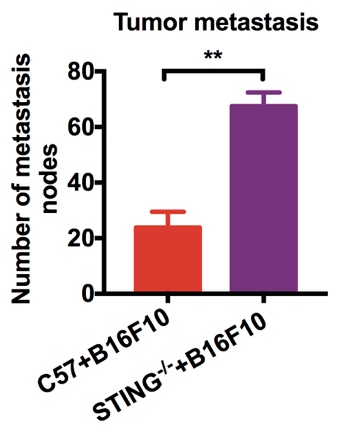


Figure S1: the metastatic foci in the lung tissues

**Figure S2: The expressions of cytokines in liver tissues.**

The expression of TNF-α, IL-2, IFN-γ and IFN-β in liver tissues was detected by RT-PCR. (^*^*P*<0.05, ^**^*P*<0.01).


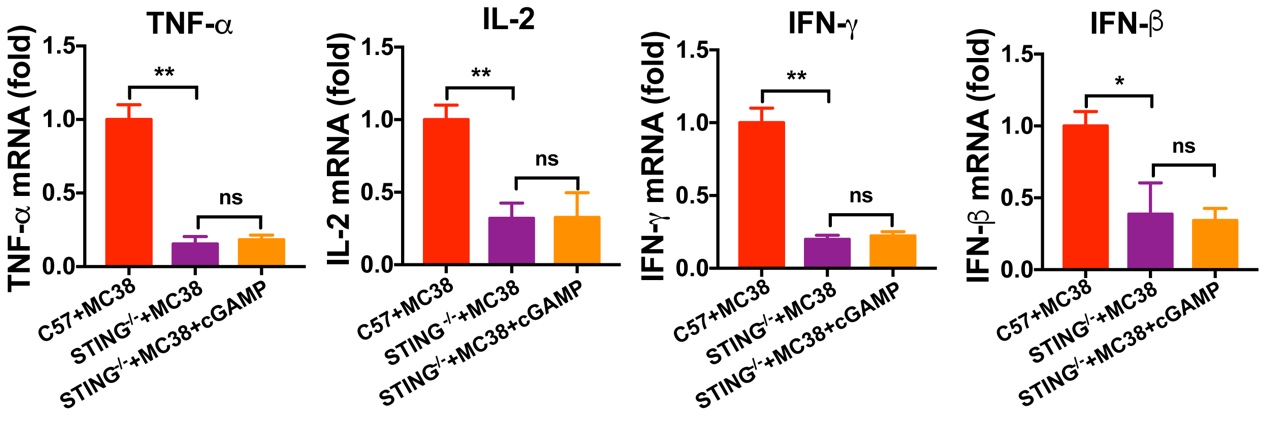


Figure S2: The expressions of cytokines in liver tissues

**Figure S3: The expression of cytokines in liver tissues of CT26 tumor bearing metastatic mice.**

The expression of cytokines in liver tissues of CT26 tumor bearing metastatic mice were quantified by RT-PCR. (^**^*P*<0.01, ^##^*P*<0.01).


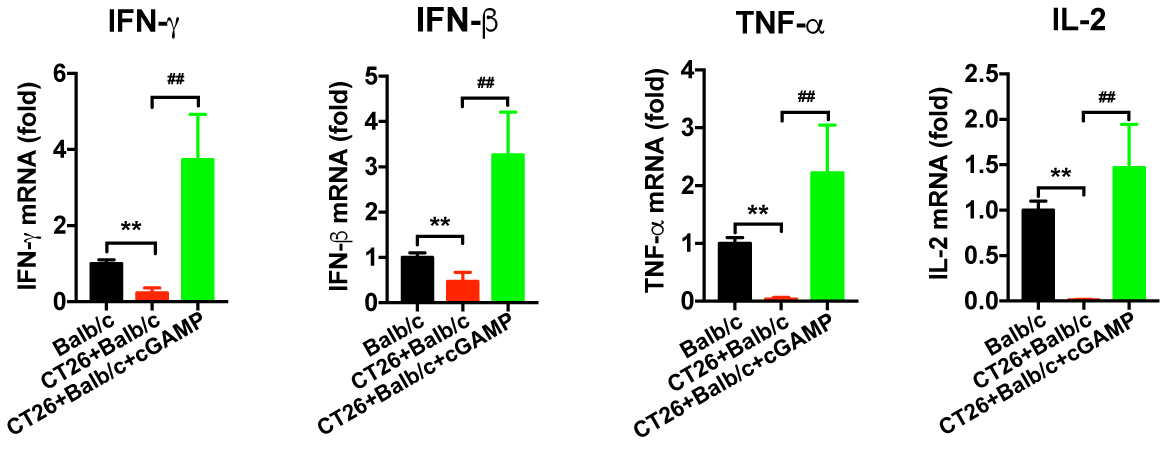


Figure S3: The expression of cytokines in liver tissues
